# Differentially expressed genes in the testes from early to mature development of banana shrimp (*Fenneropenaeus merguiensis*)

**DOI:** 10.1371/journal.pone.0292127

**Published:** 2023-10-09

**Authors:** Uraipan Saetan, Wilaiwan Chotigeat

**Affiliations:** Molecular Biotechnology and Bioinformatics Program, Division of Biological Science, Faculty of Science, Prince of Songkla University, Hat Yai, Songkhla, Thailand; Shanghai Ocean University, CHINA

## Abstract

Banana shrimp (*Fenneropenaeus merguiensis*) is an economically important species in Thailand owing to the high value of globally exported frozen brine shrimps. However, the regulatory mechanisms governing spermatogenesis and testicular development in this species are poorly understood. High-throughput RNA sequencing was used to investigate the mechanisms and regulated genes involved in testis development using transcriptome profiling of juvenile and adult banana shrimp testes. Differentially expressed genes (DEGs) in these two libraries were identified and quantified to confirm gene expression. DEGs were found in 7,347 genes, with 4,465 upregulated and 2,882 downregulated. Some of these genes were designated as candidate genes, and six specific DEGs, including *PRM1*, *SPATA20*, *Sry*, *SSRF*, *Sxl*, and *Tra-2c*, were selected to confirm the reliability of the RNA-seq data using qPCR. Moreover, six non-DEGs were chosen based on testis-specific and regulatory genes that support a specific function in spermatogenesis and testis development in this species, including *Dsx*, *Gfra2*, *IAG*, *Sox9*, *Sox13*, and *Sox14A*. Furthermore, *Sry*, *Sox14A*, *Sox14B* and *SPATA20* were identified in early stages (nauplius-postlarvae) of shrimp development to provide more information involving testes formation and development. The transcript data from this study could differentiate a group of genes required at the early and late stages of testis development and both sets of testis development. Therefore, this information would help in manipulating each stage of testicular development.

## Introduction

Penaeid shrimp testes contain a testicular lobule where spermatogenesis occurs. Spermatogenesis is the process of haploid spermatozoa production and is divided into two stages: spermatogenesis and spermiogenesis. Spermatogenesis is the process of diploid spermatogonia and haploid spermatid production, and spermiogenesis is transforming mature spermatids into spermatozoa [1]. Many studies in crustaceans have focused on spermiogenesis because the most representative process data are available for each species, including *Sicyonia ingentis* [2], *Parapenaeus longirostris* [3], *Macrobrachium rosenbergii* [4], *Fenneropenaeus chinensis* [5], *Litopenaeus vannamei* [6], and *Penaeus monodon* [7] However, spermatogenesis in *Fenneropenaeus merguiensis* is still unknown.

*F*. *merguiensis* is widely distributed in tropical and subtropical waters, especially on the northern coast of Malaya, the west coast of the Philippines, Indonesia, Australia, and Thailand. This species has been observed to mature at around 6–7 months, with a slight difference between males and females [8]. Owing to its high commercial importance in many areas, such as the Persian Gulf and Pakistan [9], the Philippines, Thailand, Indonesia, and Australia, a better understanding of its reproductive development, especially spermatogenesis and testis development, is necessary to establish the high quality and number of spermatozoa produced.

Previous transcriptome analyses using RNA-seq have been conducted in many shrimps to improve broodstock reproduction for industrial farming. Transcriptomic data from shrimp testes have been widely reported; for example, the gene expression profile of *Macrobrachium nipponense* testes was analyzed to identify candidate genes involved in male reproduction. *Fem-1*, *Fem-2*, and *Fem-3* promote male development in the sex-determining protein complex [10]. The de novo transcriptome of the Japanese mantis shrimp (*Oratosquilla oratoria*) was also examined. Some regulated gene expression in males has been identified, including cathepsin I and cathepsin D-like [11]. The transcriptome of *P*. *monodon* was recently studied, and various organs, particularly the testes, were examined, providing helpful information for gene expression profiles involved in testis regulation [12].

Several regulatory genes involved in testis differentiation and maturation have been studied; for example, *cyclin A* and *cyclin B* [13], cell division cycle 2 (*Cdc2*) [14], mitogen-activating protein kinase 1 (*MAPK1*) [15] in *P*. *monodon*; activated protein kinase C1 (*RACK1*) [16], cell apoptosis susceptibility (*FcCAS*) [17] in *F*. *chinensis*; *vasa-like* [18], spermatogonial stem-cell renewal factor *(SSRF)*, spermatogenesis-associated proteins (*SPATA2*), sperm protamine P2 (*PRM2*), testis-specific serine proteases (*TESSP*) in *L*. *vannamei* [19], and Doublesex and Mab-3 Related Transcription Factor 1 (*Dmrt1*) in *Palaemon serratus* [20].

Currently, the regulated genes involved in testicular development and spermatogenesis in *F*. *merguiensis* are poorly understood. The transcriptomic profiles of juvenile and mature *F*. *merguiensis* testes were studied for candidate regulatory genes and involved pathways using high-throughput RNA-seq technology. The candidate genes in testis development were selected from the ≥log 2-fold change in the compared stages, and the expression of the selected candidate genes was confirmed using qPCR. The outcome of this study provides crucial information on the regulatory gene expression involved in banana shrimp testis development.

## Materials and methods

### Ethics declarations

This study was approved and conducted according to the Institutional Animal Care and Use Committee (IACUC) of the Prince of Songkla University.

### Sample collection

Juvenile and adult male shrimp were purchased from the Trang and Nakhon Si Thammarat provinces, respectively, of Thailand. The shrimp were reared from postlarva day 5 until the testis began to form an external male sex organ were classified as juvenile, approximately 6–7 cm in length and 1.5–2.0 g in weight (about postlarva day 114^th^), whereas adult shrimp were wild-caught, about 16–17 cm in length, and 28.0–30.0 g in weight. Three male shrimp from each group were dissected to harvest the testes. The excised testes were divided into two pieces. One piece was fixed in Davidson’s solution for histological examination, whereas the others were immersed immediately in TRIzol reagent (GIBCO BRL, Grand Island, NY, USA) for RNA preparation.

### Histological analysis of testis samples

Testes fixed in Davidson’s solution were processed and embedded in paraffin wax. The samples were cut into 5 μm thick sections and stained with hematoxylin and eosin to examine the histological structure of juvenile and adult shrimp testes. An Olympus BX51 microscope (Tokyo, Japan) with 40X objectives was used to capture images of all samples.

### RNA isolation and cDNA library construction

Total RNA was isolated from each sample using TRIzol reagent (GIBCO BRL), following the manufacturer’s instructions. Potentially contaminated genomic DNA was removed using RQ1 RNase-free DNase (Promega, Madison, WI, USA). The RNA concentration and quality were determined using an Agilent 2100 Bioanalyzer (Agilent RNA 6000 Nano Kit, Agilent, Santa Clara, CA, USA). For the construction of cDNA libraries, three RNA samples from each group were pooled. The first step in the library construction process is mRNA enrichment via oligo dT selection or rRNA depletion. RNA samples were fragmented in the second step before being reverse-transcribed to double-stranded cDNA using the N6 random primer. Before 3′-adenylation, the synthesized cDNA was repaired. Adaptors were then ligated to 3′-adenylated cDNA ends. The ligation products were purified, followed by an amplification step to enrich the purified cDNA template. The PCR products were denatured using heat, and the single-stranded DNA was cyclized using splint oligo-and DNA ligase. Each sample library was sequenced on the BGISEQ-500 platform at Beijing Genomics Institute (BGI, Shenzhen, China). The raw data for the assembled transcriptome was submitted to the Sequence Read Archive (SRA) under the accession number PRJNA961319.

### De novo assembly and functional annotation

Following sequencing, low-quality reads (more than 20% of base qualities were less than 10), reads with adaptors, and reads with unknown bases (N bases greater than 5%) were filtered using SOAPnuke to obtain clean reads. Clean reads were assembled into unigenes using Trinity (v2.0.6) (https://github.com/trinityrnaseq/trinityrnaseq/releases/tag/v2.0.6). Using the TGICL software (v2.0.6) (https://sourceforge.net/projects/tgicl/files/tgicl/), gene family clustering was performed to obtain the final unigenes. The unigenes were then aligned to NT, NR, Clusters of Orthologous Group (COG), and SwissProt databases to perform functional annotation using BLAST (v2.2.23) and Diamond (v0.8.31) software (https://github.com/bbuchfink/diamond). The Gene Ontology (GO) annotation was performed using Blast2GO (v2.5.0), providing biological functions at the molecular, cellular, and tissue system levels. Pathway enrichment analysis was performed using the online KEGG Automatic Annotation Server (KAAS) (http://www.genome.jp/kegg/kaas/) to understand the high-level functions of metabolic pathways.

### Differential expression genes (DEGs) analysis

Based on the assembly results, all of the clean reads from each sample were mapped to unigenes using Bowtie2 software (v2.2.5) and the gene expression level was calculated using RSEM (v1.2.12). The relative expression of a transcript was calculated by counting fragments per kilobase of transcript per million mapped reads (FPKM). A Poisson distribution was performed to identify differentially expressed genes (DEGs) between juvenile and adult shrimp testes, with the false discovery rate (FDR) adjusted to ≤ 0.001. A fold change ≥ 2 with the log2 ratio of DEGs between the adult testis and juvenile testis (adult testis/juvenile testis) ≥ 1 was used to judge the significance of DEGs.

### Quantitative real-time PCR (qRT-PCR) validation

Genes involved in testis development were selected using the KEGG pathway annotation, including six specific DEGs; Sperm Protamine P1-like (*PRM1*), Spermatogenesis-associated protein 20 (*SPATA20*), Sex-determining region Y (*Sry*), *SSRF*, Sex-lethal (*Sxl*), and Transformer-2c (*Tra-2c*), as well as six non-DEGs; Doublesex (*Dsx*), GDNF family receptor alpha 2 (*Gfra2*), Insulin-like androgenic gland hormone (*IAG*), Sry-box transcription factor 9 (*Sox9*), Sry-box transcription factor 13 (*Sox13*), and Sry-box transcription factor 14A (*Sox14A*). Gene expression data from RNA-seq were validated using qRT-PCR. Briefly, total RNA from juvenile (n = 3) and adult shrimp (n = 3) testes were isolated and pooled equally, and reverse transcription was performed to synthesize cDNA using AMV reverse transcriptase (Promega) according to the manufacturer’s protocol. qRT-PCR was performed with three technical replicates in the Mx3000P qPCR system (Stratagene, San Diego, CA, USA) using the FastStart Universal SYBR Green Master (Roche, Germany). *β*-actin was used as an endogenous reference to eliminate sample-to-sample variations and normalize changes in specific gene expression. The amplification procedure was as follows: pre-denaturation at 94 °C for 3 min followed by 40 cycles of denaturation at 94 °C for 30 s, annealing at the required temperature for 30 s, and extension at 72 °C for 45 s. The relative expression levels of the DEGs were calculated using the 2^−ΔΔCT^ method, and data were expressed as mean ± SD.

### Analysis of gene expression involved in testes development

Some genes involved in testes development, including *SPATA20*, *SOX14A*, *SOX14B*, and *Sry* were examined for expression in different stages of shrimp, including nauplius, zoea, postlarva (PL) 5, 10, 20, 30, 50, 70, 90, and adult shrimp (about 5–6 months old). RNA was extracted from whole shrimp from nauplius to PL90, while only testes from adult shrimp were collected. The cDNA was then converted using AMV Reverse Transcriptase (Promega, Madison, WI, USA) and amplified for each gene. The condition for PCR was performed as previously described in qPCR. The expression of all genes was examined using 8% polyacrylamide gel electrophoresis.

## Results

### Histological characteristics of juvenile and adult testis

**Fig 1:**
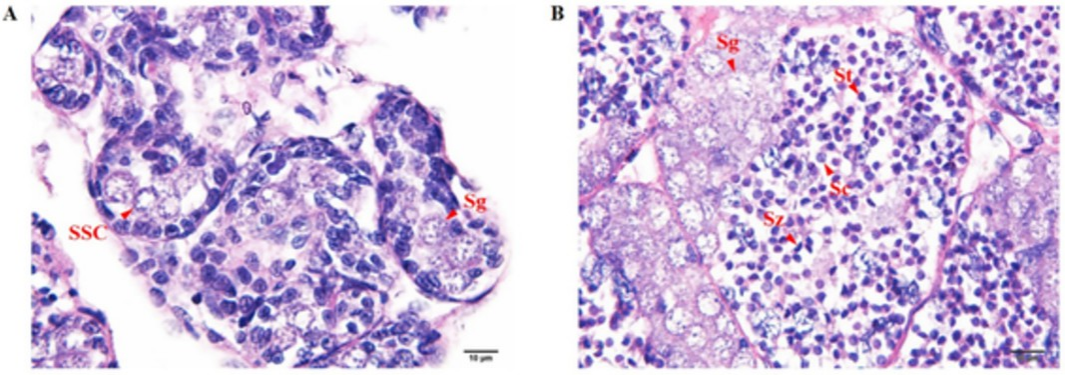
Histological analysis of the juvenile and adult testes. The histological analysis of the juvenile testis (A) and adult testis (B) showed various cell types in the lobules of the testes. SSC, Sg, Sc, St, and Sz indicated somatic stem cells, spermatogonia, spermatocyte, spermatid, and spermatozoa, respectively.

During the process of spermatogenesis, histological examination revealed that sperm developed into several stages, including spermatogonia (Sg), spermatocyte (Sc), spermatid (St), and spermatozoa (Sz). Spermatogonia (Sg) are cells found in the seminiferous tubules with a pale and rounded nucleus containing chromatin. They were the spermatogenic cells attached to the basal lamina of the seminiferous tubule and were engaged by the surrounding of somatic stem cell (SSC). After mitosis division, Sg was divided and transformed into Sc, a medium-sized cell with a slightly condensed spherical nucleus. Then, Sc was divided into St with a smaller and more condensed spherical nucleus after the second meiotic division. High-condensed chromatin was appeared during the maturation of St into Sz, and the nucleus becomes a distorted sickle shape and darkens. The difference in morphological appearance between juvenile and adult testes was classified. The juvenile testis (Fig 1A) contained more Sg cells than the other types, whereas the adult testis (Fig 1B) contained multiple stages of sperm and was primarily St and Sz. In addition, adult testis had more total cells than juvenile testis.

### mRNA expression profiles of the juvenile and adult testes

Genes involved in the development of banana shrimp testes were identified by constructing two juvenile and adult testis libraries using the BGISEQ-500 platform. More than 77.13 and 80.63 million raw reads of juvenile and adult testis were generated. After low-quality, adaptor-polluted, and high content of unknown base (N) read, they were filtered, and the total clean reads of juveniles and adults were obtained with 69.25 and 70.18 million reads, respectively (Table 1). Trinity software was used to perform de novo assembly with clean reads, and TGICL software was used to remove abundance and obtain unigenes from cluster transcripts.

**Table 1:** Sequencing statistics analysis of juvenile and adult testes transcriptome.

Sample	Juvenile testis	Adult testis
**Sequencing results**		
Total Raw Reads (M)	77.13	80.63
Total Clean Reads (M)	69.25	70.18
Clean Reads Ratio (%)	89.78	87.04
Clean Reads Q20 (%)	96.53	96.83
Total unigene number with N50	38,216 with 2,295	40,789 with 2,610
**Alignment results**		
Total Bases	10,387,722,900	10,526,715,600
Total Reads	69,251,486	70,178,104
Total Mapped Reads	58,811,734	58,787,076
Unique Mapped Reads	36,037,600	34,150,556
Mapping Ratio (%)	84.92	83.77
**Annotation results**		
NR	24,290 (45.71%)	
NT	16,566 (31.17%)	
SwissProt	19,612 (36.91%)	
KEGG	20,203 (38.02%)	
COG	17,993 (33.86%)	
GO	7,324 (13.78%)	
InterPro	16,194 (30.47%)	

### DEGs annotation and pathway analysis

**Fig 2:**
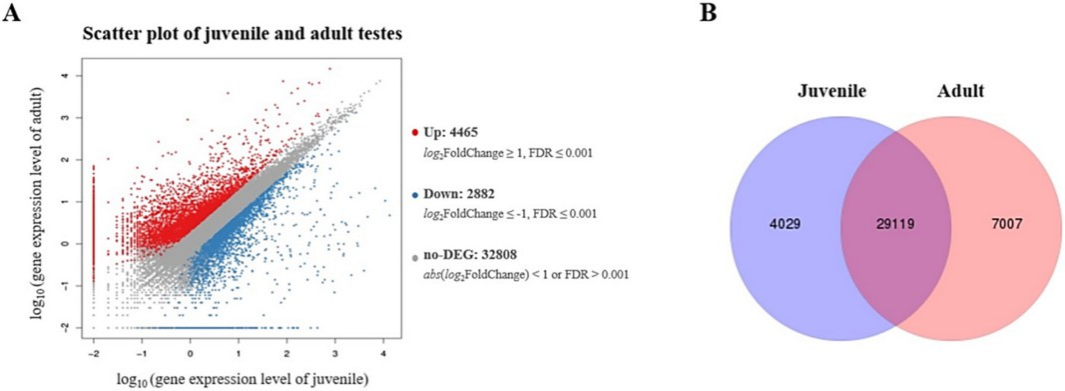
Summary of DEG plot. A Scatter plot of DEGs (A) shows the upregulated genes with red dots, down-regulated genes with blue dots, and no-DEGs with grey dots. The vertical axis represents the log_10_ of the expression level in the adult testis gene, whereas the horizontal axis represents the log_10_ of the juvenile testis gene expression level. Venn diagram (B) of gene expression was analyzed between juvenile and adult testes.

The expression levels of all genes were detected using PossionDis Algorithm to verify the DEGs between the juvenile and adult testes. A Scatter plot, Venn diagram, and heatmap plot were generated to show the distribution of the DEGs. In total, 7,347 genes were identified as DEGs, of which 4,465 were upregulated, and 2,882 were downregulated (Fig 2A). A Venn diagram indicates the number of particular expression genes in one sample and the number expressed in both models (Fig 2B). In addition, gene expression was higher in adult testes (7,007 genes) than in juvenile testes (4,029 genes).

**Fig 3:**
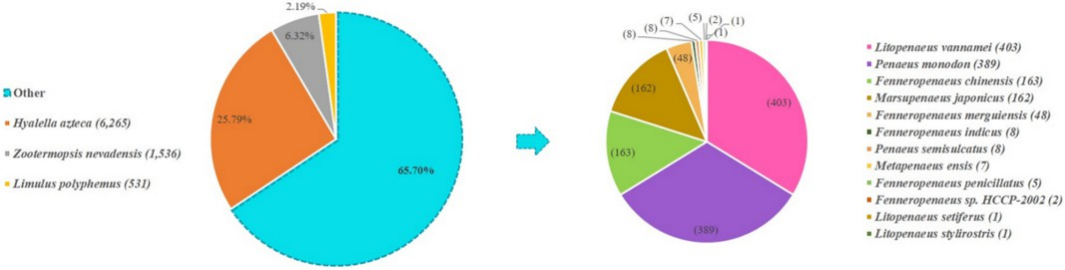
The homologous species distribution is annotated against the NR database. The number showed the percentages of the best blast hits of unigenes among other species.

The result of NR annotation was investigated for species distribution to evaluate the evolutionary conservation. A total of 24,290 genes were mapped to the NR database using BLASTX. The top three species with the most abundant matched transcripts are *Hyalella Azteca* (25.79%), *Zootermopsis nevadensis* (6.32%), and *Limulus Polyphemus* (2.19%), all of which are members of the Arthropoda phylum, to which *F*. *merguiensis* also belongs. Among others abundant in the Penaeidae family, *Litopenaeus vannamei* was the most abundant shrimp (1.66%), followed by *P*. *monodon* (1.60%) and *F*. *chinensis* (0.67%) (Fig 3).

**Fig 4:**
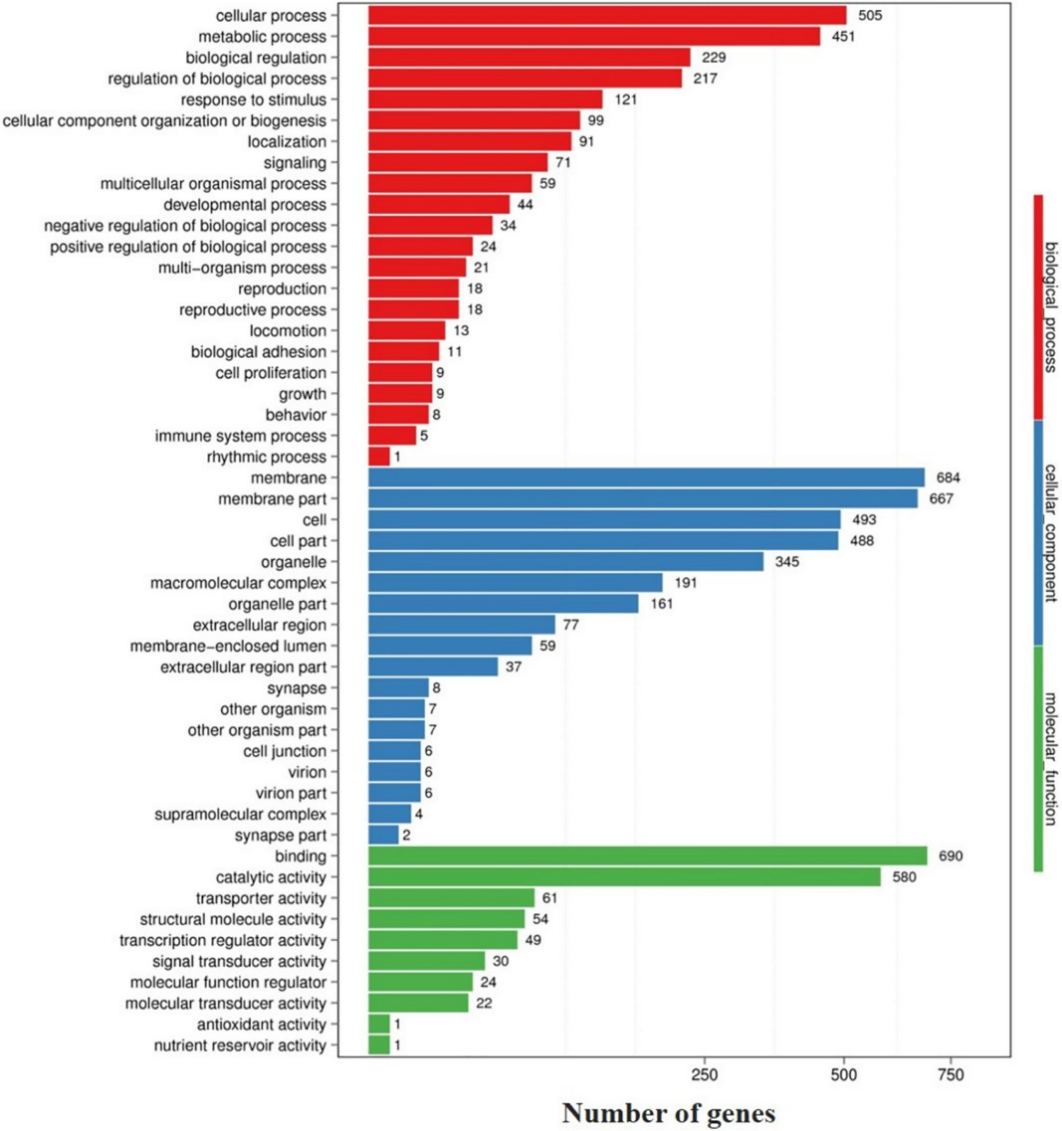
Bar plot of enriched GO terms of DEGs. The vertical axis represents the name of each Go term, whereas the horizontal axis represents the number of genes. Each color refers to the different terms of GO.

Gene ontology (GO) analysis of DEGs was classified for functional enrichment based on three ontologies: biological process, cellular component, and molecular function (Fig 4). A total of 22 GO terms belonged to the biological process category, which was mainly focused on cellular processes (505 genes), metabolic processes (451 genes), and biological regulation (229 genes). For cellular components, approximately 18 GO terms were members of "membrane" (684 genes), "membrane part" (667 genes), and "cell" (493 genes). The molecular function was the last ontology, which included 10 GO terms. The top three counts were "binding" (690 genes), "catalytic activity" (580 genes), and "transporter activity" (61 genes).

In total, 20,203 genes with KEGG pathway annotations were mapped to 332 pathways. The most prominent pathway member was the metabolic pathway, which included 2,481 genes (12.28%). However, only 4,703 genes were identified as DEGs. The pathway classification of DEGs has been classified into seven categories: metabolism, genetic information processing, environmental information processing, cellular processes, organismal systems, human diseases, and drug development. Biological pathways involved in the testis development of *F*. *merguiensis* were discovered, including the GnRH signaling pathway, insulin signaling pathway, Wnt signaling pathway, Notch signaling pathway, aldosterone synthesis and secretion, TGF-beta signaling pathway, and thyroid hormone signaling pathway.

### DEGs involved in spermatogenesis and testis development

Spermatogenesis is the process of haploid spermatozoa production. The discovery of marker genes for this process will improve our understanding of the regulatory processes that govern spermatogenesis. Some functional genes involved in late spermatogenesis were upregulated in the adult shrimp testis compared to the juvenile shrimp testis, including Argonaute 4 (*AGO4*), Crustacean hyperglycemic hormone (*CHH*), Feminization-1b (*Fem-1b*), Insulin receptors (*Irs*), Nanos (*Nos*), Paired Box 7 (*PAX7*), *PRM1*, *SPATA20*, *SSRF*, and *Tra-2*, while others were downregulated, including *Sxl*, *Sry*, and *Tra-2c* (Table 2, S1 Table).

**Table 2:** Some functional genes involved in spermatogenesis and testis development.

Gene	Description	log2FoldChange (adult/juvenile)	*P-value*	FDR	Up/Down
*AGO4*	Argonaute 4	1.562	0.00E+00	0	Up
*CHH*	Crustacean hyperglycemic hormone	3.665	0.00E+00	0	Up
*Dmrt1*	Doublesex- and mab-3-related transcription factor 1	−4.644	5.65E-02	1.21E-01	-
*Dsx*	Doublesex	5.459	5.20E-01	6.49E-01	-
*Fem-1b*	Feminization-1b	1.065	3.20E-05	1.22E-04	Up
*IAG*	Insulin-like androgenic gland	2.585	2.65E-01	3.98E-01	-
*IGFBP*	Insulin-like growth factor binding protein	0.954	2.00E-304	2.29E-302	-
*Irs*	Insulin receptors	5.248	7.87E-12	5.06E-11	Up
*Nos*	Nanos	1.126	6.17E-06	2.55E-05	Up
*PAX7*	Paired Box 7 binding protein 1	2.492	2.51E-46	4.55E-45	Up
*PRM1*	Sperm Protamine P1-like	4.755	2.67E-15	2.05E-14	Up
*Sox13*	SRY-related HMG-box containing transcription factor 13	1.281	4.37E-02	9.62E-02	-
*Sox14A*	SRY-related HMG-box containing transcription factor 14A	0.867	4.87E-01	6.19E-01	-
*Sox14B*	SRY-related HMG-box containing transcription factor 14B	0.347	4.17E-03	1.18E-02	-
*SPATA20*	Spermatogenesis-associated protein 20	2.158	1.42E-07	6.80E-07	Up
*SSRF*	Spermatogonia stem-cell renewal factor	1.520	6.33E-06	2.61E-05	Up
*Sxl*	Sex-lethal	−2.388	0.00E+00	0	Down
*Tra-2*	Transformer-2	1.322	3.81E-82	1.17E-80	Up
*BMP7*	Bone morphogenetic protein 7	−0.240	3.65E-01	5.21E-01	-
*Cdc2*	Cell division cycle 2	−0.437	1.61E-24	1.76E-23	-
*GEM*	Gem-associated Protein 2-like Isoform X1	−0.564	8.07E-12	5.19E-11	-
*Gfra2*	GDNF Family Receptor Alpha 2	−0.336	4.65E-02	1.01E-01	-
*GnRHr*	Gonadotropin-releasing hormone receptor	−1.271	3.12E-01	4.58E-01	-
*HSP90*	Heat shock protein 90	−0.496	0.00E+00	0.00E+00	-
*Pcna*	Proliferating cell nuclear antigen	−0.755	4.76E-78	1.39E-76	-
*Sox5*	SRY-related HMG-box containing transcription factor 5	−0.501	4.18E-04	1.39E-03	-
*Sox6*	SRY-related HMG-box containing transcription factor 6	−0.209	4.26E-02	9.40E-02	-
*Sox8*	SRY-related HMG-box containing transcription factor 8	−0.186	9.56E-01	9.85E-01	-
*Sox9*	SRY-related HMG-box containing transcription factor 9 (promoter region)	−7.066	2.35E-01	3.86E-01	-
*SPATA5*	Spermatogenesis-associated protein 5	−0.923	1.99E-22	2.05E-21	-
*Sry*	Sex-determining region Y	−2.445	7.19E-05	2.64E-04	Down
*Tra-2c*	Transformer-2c	−1.346	8.47E-13	5.74E-12	Down
*VASA*	DEAD box family of RNA helicases	−0.363	1.18E-89	3.97E-88	-

In addition, several genes had a trend of increased expression, such as *Dsx*, *IAG*, Insulin-like growth factor binding protein (*IGFBP*), *Sox13*, *Sox14A*, and *Sox14B*. In contrast, genes had a trend to reduce expression during the testes development, including Bone morphogenetic protein 7 (*BMP7*), *Cdc2*, Gem-associated Protein 2-like Isoform X1 (*GEM*), *Gfra2*, Gonadotropin-releasing hormone receptor (*GnRHr*), Heat shock protein 90 (*HSP90*), Proliferating cell nuclear antigen (*Pcna*), SRY-related HMG-box containing transcription factor 5 (*Sox5*), SRY-related HMG-box containing transcription factor 6 (*Sox6*), SRY-related HMG-box containing transcription factor 8 (*Soc8*), *Sox9*, Spermatogenesis-associated protein 5 (*SPATA5*), and DEAD box family of RNA helicases (*VASA*). Although these genes were not significantly differentially expressed between juvenile and mature testes (*P>0*.*05*), the results indicated that the expression of these genes is essential in both the juvenile and mature stages of testes development.

### Validation of RNA-Seq data by qPCR

**Fig 5:**
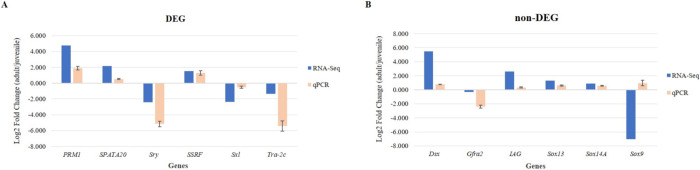
Validation of RNA-Seq using qPCR. The expression of the selected DEGs (A) and non-DEGs (B) from RNA-Seq data was compared to qPCR in the fold change of adult/juvenile testis. The log2FC values of qPCR are shown as the means ± SD.

qPCR was used to clarify the expressed genes in the transcriptome data. The male-specific gene was selected from Table 2, including upregulated genes such as *PRM1*, *SPATA20*, and *SSRF*, as well as downregulated genes such as *Sry*, *Sxl*, and *Tra-2c* (Fig 5A). Moreover, non-significantly different expressed genes, including *Dsx*, *Gfra2*, *IAG*, *Sox9*, *Sox13*, and *Sox14A* were also chosen for validation (Fig 5B). The majority of them corresponded with transcriptome data, with the exception of *Sox9*, which was a +1-fold change determined by qPCR and a -7-fold change determined by transcriptome analysis.

#### Expression of genes involved in male sex differentiation and testes development

Some selected genes such as *SPATA20*, *Sox14A*, *Sox14B*, and *Sry* were identified from each stage of shrimp from nauplius to adult shrimp to demonstrate the potential function of genes involved in male sex differentiation and testes development. The presence of these genes at an early stage, as nauplius suggested that they may play an important role in male-sex differentiation and development, however, this needs to be investigated further using other methods, such as gene knockdown or knockout, to determine the true effect of these genes’ defection on sperm synthesis.

## Discussion

This transcriptome data analysis between juvenile and adult testis revealed mRNA expression profiles of the juvenile and adult testes that specific gene expression was required for each stage. The upregulated gene expression for spermatogenesis in adult testis, for example, *SPATA20*, *SSRF*, *Nos*, *PRM1* (Table 2, S1 Table). *SPATA*s have been identified as testis-specific genes that regulate apoptosis during zebrafish spermatogenesis [21]. The upregulation of *SPATA20* was greater in adult testes than in juvenile testes in both transcriptome and qPCR analyses in this study. Furthermore, its expression was found to be consistent from larva to adult, supporting the testis development. Other *SPATA*s genes with testis-biased expression patterns were discovered, including *SPATA5L1*, *SPATA6*, and *SPATA18*, but there was no difference between juvenile and adult testes. However, *SPATA2*, *SPATA5*, and *SPATA20* were found to be essential genes due to their expression was detected in the later stages of spermatogenesis in *L*. *vannamei* [19]. Interestingly, the expression of *SPATA20* was examined and found from the early stage at nauplius to the adult shrimp, suggesting that this gene may be involved in the process of sperm differentiation and formation, as the lack of this gene in mice resulted in sperm number reduction and abnormal of sperm morphology [22]. Spermatogonia stem cells are known to play a role in self-renewal and sperm differentiation [23]. Many investigations of *SSRF* were reported in mammals, but there have been few findings in crustaceans [24–26]. The high expression of *SSRF* allows for the renewal of the spermatogonia number in the adult testis. *SSRF* is named after its role in self-renewing cells in male fertility spermatogenesis, which allows stem cell populations to proliferate [25]. Therefore, the expression of *SSRF* was increased in the adult testes rather than in juvenile testes in this study, indicating that it plays a role in testicular development. In addition, *Nos* genes express the conserved zinc-finger RNA-binding proteins that play roles in maintaining germline stem cell function [27–29]. *Nos* gene expression was found to be higher in the adult testes than in the juvenile testes to maintain germ cells in the adult. *PRM1*, which involves sperm development, also increased expression in the adult testis of this study. *SSRF* and *PRM1* expression results relate to the histological of the adult testis (Fig 1), which showed a lot of spermatogonia population and spermatozoa were found.

*Fem-1b* was upregulated in the adult testis of *F*. *merguiensis*, which indicated a requirement for spermatogenesis. In addition, *Fem-1b*, a homolog of *Fem-1*, was found to have high expression in early embryonic development and in the testis, ovary, hepatopancreas, and muscles of *E*. *sinensis* [30, 31]. Therefore, *Fem-1b* was suggested as a maternal gene expression during the early embryonic stage, and it is required during the late stages of gonadal development [30]. The result of this study corresponded with the detection of *Fem-1* in spermatogonia of *P*. *vannamei* [32]. This study also showed that high *Sxl* expression was required for juvenile testis development. *Sxl* was studied in several decapods, such as the Redclaw crayfish: *C*. *quadricarinatus* [33], *E*. *sinensis* [34] and *P*. *vannamei* [35]. They suggested that *Sxl* does not involve in sex determination, but it is required for embryonic development, sexual differentiation, and gonad development.

*PAX7*, *AGO4*, *CHH*, and *Irs* also upregulated genes that supported the development of adult testes in this study. As previously reported in mice, *PAX7* is present in the testes at birth and plays a role in spermatogenesis, particularly in the spermatogonial stem cell population [36]. *AGO4* encodes a protein that contains PAZ and PIWI domains and plays gene regulation via RNA interference and short-interfering-RNA-mediated gene silencing [37]. A knockdown study of this gene in *P*. *monodon* [38] using RNAi found that reducing this gene in transcript level resulted in testicular maturity and a decrease in the number of spermatogonia in the spermatophore, revealing the critical role in controlling spermatogenesis.

Generally, *CHH* is known to elevate circulating glucose under stressful conditions and has multiple functions, such as ecdysteroidogenesis, osmoregulation, and vitellogenesis. Some *CHH* neuropeptides were suggested to have an extra function in reproduction at the adult stage [39]. The function involved in testis development was discovered while studying this gene in *M*. *nipponense* [40]. *CHH* was found significantly upregulated to provide energy (glucose) for adult testes development and corresponded with upregulated *Irs* expression for importing glucose into cells (Table 2).

In contrast, *Tra-2c* significantly down expression in the adult testis. The *Tra-2* gene has been examined in various crustaceans, including *P*. *monodon* [41], *F*. *chinensis* [42], *M*. *nipponense* [43], and *C*. *quadricarinatus* [44]. Although the nucleotide sequences are similar to *D*. *melanogaster* [45], the alternative splicing patterns are quite different, implying a different mechanism of sex differentiation between crustaceans and insects, which is dependent on the alternative splicing patterns of the pre-mRNA of genes involved in sex determination. Three isoforms of *Tra-2* have been identified in *F*. *chinensis*: *Tra-2a*, *Tra-2b*, and *Tra-2c*. The transcript of *Tra-2c* was significantly increased at the mysis stage and showed a significantly higher expression level in females than in male. Therefore, *Tra-2c* was suggested to be involved in the female sex determination of *F*. *chinensis* [42]. In this transcriptome, *Tra-2c* was more abundant in juvenile testes than in adult testes. This phenomenon may be explained by the fact that this gene may require in early testicular development, and the expression level in the larval stage or postlarval stage is interesting to be detected for clarification of *Tra-2c* in female sex determination in *F*. *merguiensis*.

**Fig 6:**
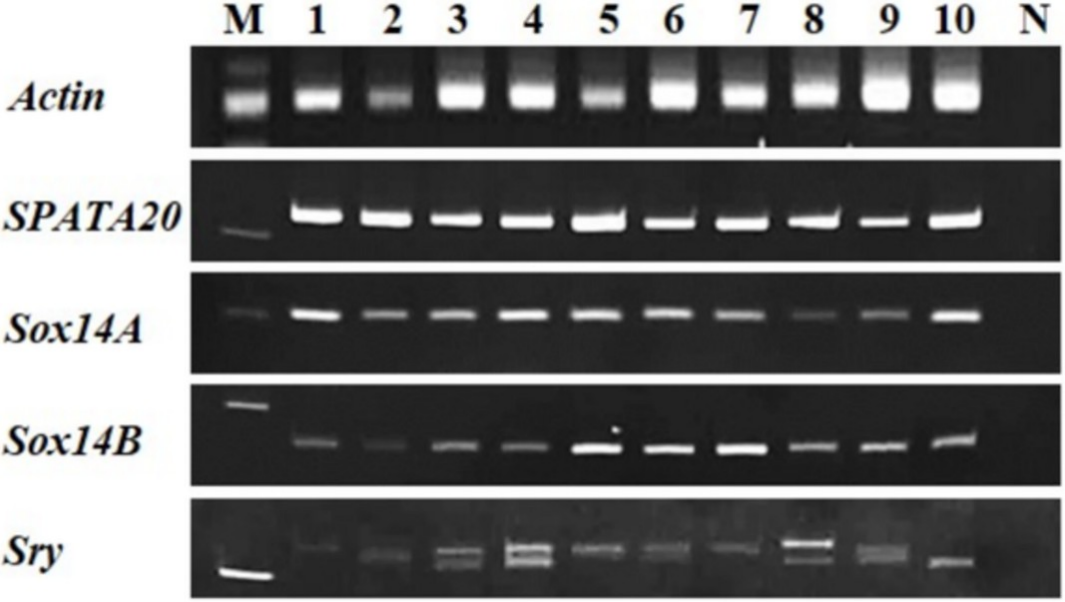
Examining the expression of genes involved in testis development included *SPATA20*, *Sox14A*, *Sox14B*, and *Sry*, at different stages in shrimp. Lane M: 100 bp DNA ladder, lanes 1–10: amplification of gene from each stage of shrimp including nauplius, zoea, postlarva 5, 10, 20, 30, 50, 70, 90, and adult, lane N: negative control containing sterile water.

The *Sox* gene family comprises a group of genes that encode a Sry-like high-mobility group (HMG) box, a male-determining gene. Many transcription factors in the *Sox* gene family play important roles in developmental processes, such as neurogenesis, sex determination and differentiation, and testis development [46]. *Sry* and *Sox9* (non-significant DEG) decreased expression in the adult testis, which means they have a role during the early testis development. *Sry* has been reported to turn off ovarian genes, whereas it turned on testicular genes, especially *Sox9*. In addition, *Sox9* turned on testicular genes and turned off ovarian genes [47]. In mice, *Sry* and *Sox9* have important roles in repressing ovarian and activating testicular differentiation genes for testis cord formation. The mechanism of the activities was reported by repressing the WNT/b-catenin transcriptional activities of ovarian-differentiating genes in a granulosa cell line [48]. In this study, the detection of *Sry* at nauplius was expressed and extended to the adult stage, implying that this gene is involved in sex determination, which was consistent with previous findings in mice that this gene begins expressed after the genital ridges first appear and remains expressed until the first morphological signs of testis differentiation become apparent [49]. Interestingly, the *Sry* gene was discovered with two approximate sizes in some stages of shrimp development (Fig 6), requiring further investigation into the real specific function to the stage of development.

*Sox* members have been found in various crustaceans, including *M*. *nipponense* [50], *P*. *vannamei* [19], and *P*. *serratus* [20]. *Sox5*, *Sox14*, and *Sox15* are found in the testes of *P*. *serratus*, with *Sox5* and *Sox14* involved in male sex differentiation, whereas *Sox15* has never been identified as a testis gene [20]. This study found *Sox5*, *Sox6*, *Sox8*, *Sox9*, *Sox13*, *Sox14A*, and *Sox14B* involved in the early stage of testis development. Since the expression of *Sox14A* and *Sox14B* was detected in the nauplius and continued into adulthood, it was hypothesized that both might be involved in male-sex differentiation and sperm development, based on previous findings in *S*. *paramamosain* that high expression of *Sox14* was reported in mature sperm stage than in spermatocyte and spermatid stages during the testis development [51]. Additionally, the highest expression of *Sox14* was detected in the fertilized egg and early embryogenesis, which had an extensive development of organs such as the optic ganglion, appendicular ganglion, and heart [52, 53], implying that *Sox14* may be involved in the embryonic development and sperm maturation and in this species.

Some of the non-DEGs found in this transcriptome, including *Dsx* and *IAG*, are known to be involved in spermatogenesis and testes development [54, 55], but after the expression was validated, upregulation of them was detected in adult testes rather than juvenile testes, indicating a potential role in testes development. In contrast, *Gfra2* was discovered in the transcriptome to be non-DEG, but its expression was found to be downregulated after the expression was investigated. According to this result, *Gfra2* may be involved in the early stages of spermatogenesis.

This transcriptome study focused on a better understanding of the regulatory genes and factors controlling spermatogenesis and testis development in *F*. *merguiensis* due to the lack of information on this species. Most of the results showed a point in the expression of the genes involved. The effect of these genes on testis development in *F*. *merguiensis* requires further investigation.

## Conclusion

Comparative transcriptome analysis of juvenile and adult testes from *F*. *merguiensis* was performed. Several candidate genes involved in spermatogenesis and testis development were identified using GO terms and biological pathways, with some differences in expression. The transcript data identified group the genes required at the early (*Sry*, *Tra-2c*, *Gfra2*, *Sox9*, and *Sxl*) and late stages (*AGO4*, *CHH*, *Fem-1*, *Irs*, *Nos*, *PRM1*, *SPATA20*, *SSRF*, *Tra-2*, *IAG*, *Sox13*, and *Sox14A*) of testis development. Therefore, this information would be useful for manipulating each stage of testicular development. In addition, some gaps were found in the knowledge about this shrimp, such as whether *Dsx* regulates testis development via *IAG* signaling and whether *Tra-2c* is involved in testis development. These results imply that further investigation of these genes will be useful in the regulation of spermatogenesis and testis development in this species.

## Supporting information

S1 Table Additional data of some functional genes involved in spermatogenesis and testis development. (DOCX)

## References

[1] Garza-Torres R, Maeda-Martínez AM, Guerrero-Tortolero DA, Obregón-Barboza H, Campos-Ramos R. Description of meiosis in female and male Pacific white shrimp *Litopenaeus vannamei* (Decapoda: Penaeidae). J Crustac Biol. 2011;31(1): 75–81. doi: 10.1651/10-3316.1

[2] Shigekawa K, Clark WH. Spermiogenesis in the Marine Shrimp, *Sicyonia ingentis* penaeidae/sperm/spermiogenesis. Dev Growth Differ. 1986;28: 95–112. doi: 10.1111/J.1440-169X.1986.00095.X 37281485

[3] Medina A. Spermiogenesis and sperm structure in the shrimp *Parapenaeus longirostris* (Crustacea: Dendrobranchiata): comparative aspects among decapods. Mar Biol. 1994;119: 449–460. doi: 10.1007/BF00347542

[4] Poljaroen J, Vanichviriyakit R, Tinikul Y, Phoungpetchara I, Linthong V, Weerachatyanukul W, et al. Spermatogenesis and distinctive mature sperm in the giant freshwater prawn, *Macrobrachium rosenbergii* (De Man, 1879. Zool Anz. 2010;249(2): 81–94. doi: 10.1016/j.jcz.2010.03.002

[5] Ge S, Wang S, Kang X, Duan F, Wang Y, Li W, et al. Transition of basic protein during spermatogenesis of *Fenneropenaeus chinensis* (Osbeck, 1765). Cytotechnology. 2011;63: 581–98. doi: 10.1007/S10616-011-9364-7 21997709 PMC3217070

[6] Alfaro-Montoya J, Braga A, Vargas M, Umaña-Castro R. Ultrastructural demonstration of the model of *Litopenaeus vannamei* (Crustacea, Penaeidae) male sexual maturation and spermatozoal capacitation. Invertebr Reprod Dev. 2017;61: 9–17. doi: 10.1080/07924259.2016.1244573

[7] Feng T, Paterson B, Johnston SD. New insights into the spermatogenesis of the black tiger prawn, *Penaeus monodon*. J Morphol. 2017;278: 689–703. doi: 10.1002/jmor.20664 28164360

[8] Hoang T, Lee SY, Keenan CP, Marsden GE. Observations on growth, sexual maturity and spawning performance of pond-reared *Penaeus merguiensis*. Aquac Res. 2002;33(11): 863–73. doi: 10.1046/j.1365-2109.2002.00726.x

[9] Holthuis LB. FAO species catalogue. Volume 1-Shrimps and prawns of the world. An annotated catalogue of species of interest to fisheries; 1980.

[10] Qiao H, Xiong YW, Jiang SF, Fu HT, Sun SM, Jin SB, et al. Gene expression profile analysis of testis and ovary of oriental river prawn *Macrobrachium nipponense*, reveals candidate reproduction-related genes. Genet Mol Res. 2015;14(1): 2041–2054. doi: 10.4238/2015.March.20.14 25867350

[11] Yan H, Cui X, Shen X, Wang L, Jiang L, Liu H, et al. De novo transcriptome analysis and differentially expressed genes in the ovary and testis of the Japanese mantis shrimp *Oratosquilla oratoria* by RNA-Seq. Comp Biochem Physiol Part D Genomics Proteomics. 2018;26: 69–78. doi: 10.1016/J.CBD.2018.04.001 29702368

[12] Pootakham W, Uengwetwanit T, Sonthirod C, Sittikankaew K, Karoonuthaisiri N. A novel full-length transcriptome resource for black tiger shrimp (*Penaeus monodon*) developed using isoform sequencing (Iso-Seq). Front Mar Sci. 2020;7: 172. doi: 10.3389/FMARS.2020.00172

[13] Visudtiphole V, Klinbunga S, Kirtikara K. Molecular characterization and expression profiles of cyclin A and cyclin B during ovarian development of the giant tiger shrimp *Penaeus monodon*. Comp Biochem Physiol Part A Mol Integr Physiol. 2009;152: 535–543. doi: 10.1016/J.CBPA.2008.12.011 19141329

[14] Phinyo M, Visudtiphole V, Roytrakul S, Phaonakrop N, Jarayabhand P, Klinbunga S. Characterization and expression of cell division cycle 2 (Cdc2) mRNA and protein during ovarian development of the giant tiger shrimp *Penaeus monodon*. Gen Comp Endocrinol. 2013;193: 103–111. doi: 10.1016/J.YGCEN.2013.07.012 23899716

[15] Ponza P, Yocawibun P, Sittikankaew K, Hiransuchalert R, Yamano K, Klinbunga S. Molecular cloning and expression analysis of the Mitogen-activating protein kinase 1 (MAPK1) gene and protein during ovarian development of the giant tiger shrimp *Penaeus monodon*. Mol Reprod Dev. 2011;78(5): 347–360. doi: 10.1002/mrd.21310 21542048

[16] Ren Q, Zhou J, Zhao XF, Wang JX. Molecular cloning and characterization of a receptor for activated protein kinase C1 (RACK1) from Chinese white shrimp; *Fenneropenaeus chinensis*. Dev Comp Immunol. 2011;35: 629–634. doi: 10.1016/J.DCI.2011.01.004 21238484

[17] Wen R, Li F, Xie Y, Li S, Xiang J. A homolog of the cell apoptosis susceptibility gene involved in ovary development of chinese shrimp *Fenneropenaeus chinensis*. Biol Reprod. 2012;86: 7–1. doi: 10.1095/BIOLREPROD.111.092635 21900679

[18] Aflalo ED, Bakhrat A, Raviv S, Harari D, Sagi A, Abdu U. Characterization of a vasa-like gene from the pacific white shrimp *Litopenaeus vannamei* and its expression during oogenesis. Mol Reprod Dev. 2007;74: 172–177. doi: 10.1002/MRD.20622 16955407

[19] Peng J, Wei P, Zhang B, Zhao Y, Zeng D, Chen X, Li M, Chen X. Gonadal transcriptomic analysis and differentially expressed genes in the testis and ovary of the Pacific white shrimp (*Litopenaeus vannamei*). BMC genom. 2015;16: 1–8. doi: 10.1186/S12864-015-2219-4 26607692 PMC4659196

[20] González-Castellano I, Manfrin C, Pallavicini A, Martínez-Lage A. De novo gonad transcriptome analysis of the common littoral shrimp *Palaemon serratus*: Novel insights into sex-related genes. BMC Genom. 2019;20: 1–5. doi: 10.1186/S12864-019-6157-4 31640556 PMC6805652

[21] Groh KJ, Schönenberger R, Eggen RI, Segner H, Suter MJ. Analysis of protein expression in zebrafish during gonad differentiation by targeted proteomics. Gen Comp Endocrinol. 2013;193: 210–220. doi: 10.1016/j.ygcen.2013.07.020 23968773

[22] Liu M, Ru Y, Gu Y, Tang J, Zhang T, Wu J, Yu F, Yuan Y, Xu C, Wang J, Shi H. Disruption of Ssp411 causes impaired sperm head formation and male sterility in mice. Biochim Biophys Acta Gen Subj. 2018;1862: 660–668. doi: 10.1016/j.bbagen.2017.12.005 29247744

[23] Ahn JS, Ryu HS, Jung SE, Shin BJ, Won JH, Um TG, et al. Expression profile of spermatogenesis associated genes in male germ cells during postnatal development in mice. J Anim Reprod Biotechnol. 2020;35: 289–296. doi: 10.12750/JARB.35.4.289

[24] Oatley JM, Brinster RL. Regulation of Spermatogonial Stem Cell Self-Renewal in Mammals. Annu Rev Cell Dev Biol. 2008;24: 263–286. doi: 10.1146/annurev.cellbio.24.110707.175355 18588486 PMC4066667

[25] Wen R, Li F, Xie Y, Li S, Zhang C, Yu K, et al. Cloning and expression analysis on a homolog of spermatogonial stem-cell renewal factor in *Fenneropenaeus chinensis*. Invertebr Reprod Dev. 2014;58: 226–234. doi: 10.1080/07924259.2014.905501

[26] Sakai M, Masaki K, Aiba S, Tone M, Takashima S. Expression dynamics of self-renewal factors for spermatogonial stem cells in the mouse testis. J Reprod Dev. 2018;64: 267–275. doi: 10.1262/jrd.2018-015 29657241 PMC6021615

[27] Wang Z, Lin H. Nanos maintains germline stem cell self-renewal by preventing differentiation. Science. 2004;303: 2016–2019. doi: 10.1126/science.1093983 14976263

[28] Draper BW, McCallum CM, Moens CB. Nanos1 is required to maintain oocyte production in adult zebrafish. Dev Biol. 2007;305: 589–598. doi: 10.1016/j.ydbio.2007.03.007 17418113 PMC1986726

[29] Lacerda SM, Costa GM, da Silva MD, Campos-Junior PH, Segatelli TM, Peixoto MT, et al. Phenotypic characterization and in vitro propagation and transplantation of the Nile tilapia (*Oreochromis niloticus*) spermatogonial stem cells. Gen Comp Endocrinol. 2013;192: 95–106. doi: 10.1016/j.ygcen.2013.06.013 23792279

[30] Song C, Cui Z, Hui M, Liu Y, Li Y. Molecular characterization and expression profile of three Fem-1 genes in *Eriocheir sinensis* provide a new insight into crab sex-determining mechanism. Comp Biochem Physiol Part B Biochem Mol Biol. 2015;189: 6–14. doi: 10.1016/J.CBPB.2015.07.003 26188322

[31] Farhadi A, Cui W, Zheng H, Li S, Zhang Y, Ikhwanuddin M, et al. The Regulatory Mechanism of Sexual Development in Decapod Crustaceans. Front Mar Sci. 2021;8: 679687. doi: 10.3389/FMARS.2021.679687

[32] Galindo-Torres P, Ventura-López C, Llera-Herrera R, Ibarra AM. A natural antisense transcript of the fem-1 gene was found expressed in female gonads during the characterization, expression profile, and cellular localization of the fem-1 gene in Pacific white shrimp *Penaeus vannamei*. Gene. 2019;706: 19–31. doi: 10.1016/J.GENE.2019.04.066 31028869

[33] Zheng J, Cheng S, Jia Y, Gu Z, Li F, Chi M, et al. Molecular identification and expression profiles of four splice variants of Sex-lethal gene in *Cherax quadricarinatus*. Comp Biochem Physiol Part B Biochem Mol Biol. 2019;234: 26–33. doi: 10.1016/J.CBPB.2019.05.002 31075502

[34] Shen H, Hu Y, Zhou X. Sex-lethal gene of the Chinese mitten crab *Eriocheir sinensis*: cDNA cloning, induction by eyestalk ablation, and expression of two splice variants in males and females. Dev Genes Evol. 2014;224: 97–105. doi: 10.1007/S00427-014-0467-Y 24549568

[35] López-Cuadros I, García-Gasca A, Gomez-Anduro G, Escobedo-Fregoso C, Llera-Herrera RA, Ibarra AM. Isolation of the sex-determining gene Sex-lethal (Sxl) in *Penaeus* (*Litopenaeus*) *vannamei* (Boone, 1931) and characterization of its embryogenic, gametogenic, and tissue-specific expression. Gene. 2018;668: 33–47. doi: 10.1016/j.gene.2018.05.024 29758296

[36] Aloisio GM, Nakada Y, Saatcioglu HD, Peña CG, Baker MD, Tarnawa ED, et al. PAX7 expression defines germline stem cells in the adult testis. J Clin Investig. 2014;124: 3929–3944. doi: 10.1172/JCI75943 25133429 PMC4153705

[37] Hutvagner G, Simard M. Argonaute proteins: key players in RNA silencing. Nat Rev Mol Cell Biol. 2008;9: 22–32. doi: 10.1038/nrm2321 18073770

[38] Ho T, Panyim S, Udomkit A. Assessment of the function of gonad-specific PmAgo4 in viral replication and spermatogenesis in *Penaeus monodon*. Dev Comp Immunol. 2021;114: 103824. doi: 10.1016/j.dci.2020.103824 32791174

[39] Chung JS, Zmora N, Katayama H, Tsutsui N. General and Comparative Endocrinology Crustacean hyperglycemic hormone (CHH) neuropeptides family: functions, titer, and binding to target tissues. Gen Comp Endocrinol. 2023;166: 447–454. doi: 10.1016/j.ygcen.2009.12.011 20026335

[40] Jin SB, Wang N, Qiao H, Fu HT, Wu Y, Gong YS. Molecular cloning and expression of a full-length cDNA encoding crustacean hyperglycemic hormone (CHH) in oriental river pawn (*Macrobrachium nipponense*). J Fish China. 2013;20: 82–92. doi: 10.3724/sp.j.1118.2013.00082

[41] Leelatanawit R, Sittikankeaw K, Yocawibun P, Klinbunga S, Roytrakul S, Aoki T, et al. Identification, characterization and expression of sex-related genes in testes of the giant tiger shrimp *Penaeus monodon*. Comp Biochem Physiol Part A Mol Integr Physiol. 2009;152: 66–76. doi: 10.1016/J.CBPA.2008.09.004 18824117

[42] Li S, Li F, Wen R, Xiang J. Identification and characterization of the sex-determiner transformer-2 homologue in Chinese shrimp, *Fenneropenaeus chinensis*. Sex Dev. 2012;6: 267–278. doi: 10.1159/000341377 22846336

[43] Wang Y, Jin S, Fu H, Qiao H, Sun S, Zhang W, et al. Molecular cloning, expression pattern analysis, and in situ hybridization of a Transformer-2 gene in the oriental freshwater prawn, *Macrobrachium nipponense* (de Haan, 1849). 3 Biotech 2019;9: 1–11. doi: 10.1007/S13205-019-1737-1 31139536 PMC6505021

[44] Cai L, Zheng J, Jia Y, Gu Z, Liu S, Chi M, et al. Molecular characterization and expression profiling of three transformer-2 splice isoforms in the redclaw crayfish, *Cherax quadricarinatus*. Front Physiol. 2020;11: 631. doi: 10.3389/FPHYS.2020.00631/FULL 32733260 PMC7363937

[45] Salvemini M, Robertson M, Aronson B, Atkinson P, Polito LC, Saccone G. Ceratitis capitata transformer-2 gene is required to establish and maintain the autoregulation of Cctra, the master gene for female sex determination. Int J Dev Biol. 2003; 53:109–120. doi: 10.1387/ijdb.082681ms 19123132

[46] Jiang T, Hou CC, She ZY, Yang WX. The SOX gene family: Function and regulation in testis determination and male fertility maintenance. Mol Biol Rep. 2013;40: 2187–2194. doi: 10.1007/s11033-012-2279-3 23184044

[47] Barrionuevo FJ, Hurtado A, Kim GJ, Real FM, Bakkali M, Kopp JL, Sander M, Scherer G, Burgos M, Jimenez R. Sox9 and Sox8 protect the adult testis from male-to-female genetic reprogramming and complete degeneration. Elife. 2016;5: e15635. doi: 10.7554/eLife.15635 27328324 PMC4945155

[48] Li Y, Zheng M, Lau YFC. The sex-determining factors SRY and SOX9 regulate similar target genes and promote testis cord formation during testicular differentiation. Cell Rep. 2014;8: 723–733. doi: 10.1016/j.celrep.2014.06.055 25088423

[49] Koopman P, Münsterberg A, Capel B, Vivian N, Lovell-Badge R. Expression of a candidate sex-determining gene during mouse testis differentiation. Nature. 1990;348: 450–452. doi: 10.1038/348450a0 2247150

[50] Hu Y, Jin S, Fu H, Qiao H, Zhang W, Jiang S, et al. Functional analysis of a SoxE gene in the oriental freshwater prawn, *Macrobrachium nipponense* by molecular cloning, expression pattern analysis, and in situ hybridization (de Haan, 1849). 3 Biotech. 2020;10: 1–10. doi: 10.1007/S13205-019-1996-X 31857938 PMC6892990

[51] Liang K, Zhang Z, Liao J, Zou Z, Wang Y. Expression analysis of Sp-Sox14 gene during embryonic and gonadal development in *Scylla paramamosain*. Journal of Fisheries of China. 2018;42: 204–215. doi: 10.11964/jfc.20170210712

[52] Fukada S, Tanaka M, Iwaya M, Nakajima M, Nagahama Y. The Sox gene family and its expression during embryogenesis in the teleost fish, medaka (*Oryzias latipes*). Development, growth & differentiation. 1995;37: 379–85. doi: 10.1046/j.1440-169X.1995.t01-3-00004.x 37282177

[53] Wang Y, Yu Y, Li S, Zhang X, Xiang J, Li F. Sex-Specific transcriptome sequencing of zoea I larvae and identification of sex-linked genes using bulked segregant analysis in Pacific white shrimp *Litopenaeus vannamei*. Marine biotechnology. 2020;22: 423–32. doi: 10.1007/s10126-020-09962-7 32281012

[54] Ye Z, Bishop T, Wang Y, Shahriari R, Lynch M. Evolution of sex determination in crustaceans. Mar Life sci Technol. 2023;5: 1–11. doi: 10.1007/s42995-023-00163-4 37073332 PMC10077267

[55] Li S, Li F, Yu K, Xiang J. Identification and characterization of a doublesex gene which regulates the expression of insulin-like androgenic gland hormone in *Fenneropenaeus chinensis*. Gene. 2018;649: 1–7. doi: 10.1016/J.GENE.2018.01.043 29339074

