# Supplementary material for: Differentially expressed genes in the testes from early to mature development of banana shrimp (Fenneropenaeus merguiensis)
Source: PLoS One. 2023 Oct 9;18(10):e0292127. doi: 10.1371/journal.pone.0292127 (PMC10561846; doi:10.1371/journal.pone.0292127)
Supplement: S1 Table — (DOCX) [file pone.0292127.s001.docx]

**S1 Table** Additional data of some functional genes involved in spermatogenesis and testis development.

| **GeneID** | **Gene** | **Description** | **Juvenile  expression** | **Adult  expression** | **log2FoldChange (adult/juvenile)** | ***P-value*** | **FDR** | **Up/**  **Down** | **Nr** |
| --- | --- | --- | --- | --- | --- | --- | --- | --- | --- |
|  |  |  |  |  |  |  |  |  |  |
| CL2902.Contig1 | *AGO4* | Argonaute 4 | 186.65 | 551.2 | 1.562 | 0.00E+00 | 0 | Up | AIE15914.1/0.0e+00/argonaute 4 [*Penaeus monodon*] |
| CL2960.Contig1 | *CHH* | Crustacean hyperglycemic hormone | 169.6 | 2152 | 3.665 | 0.00E+00 | 0 | Up | AFD32402.1/1.1e-37/hyperglycemic hormone 1-like protein [*Fenneropenaeus chinensis*] |
| Unigene27848 | *Dmrt1* | Doublesex- and mab-3-related transcription factor 1 | 0.25 | 0.01 | −4.644 | 5.65E-02 | 1.21E-01 | - | ARK36620.1/3.2e-17/iDmrt1 [*Sagmariasus verreauxi*] |
| Unigene20125 | *Dsx* | Doublesex | 0.01 | 0.44 | 5.459 | 5.20E-01 | 6.49E-01 | - | Not available |
| Unigene11923 | *Fem-1b* | Feminization-1b | 1.74 | 3.64 | 1.065 | 3.20E-05 | 1.22E-04 | Up | ANN47504.1/1.0e-247/Fem1b [*Macrobrachium nipponense*] |
| Unigene29000 | *IAG* | Insulin-like androgenic gland | 0.01 | 0.06 | 2.585 | 2.65E-01 | 3.98E-01 | - | AFU60546.1/4.9e-83/insulin-like androgenic gland hormone isoform 2 [*Fenneropenaeus chinensis*] |
| CL212.Contig2 | *IGFBP* | Insulin-like growth factor binding protein | 44.39 | 85.98 | 0.954 | 2.00E-304 | 2.29E-302 | - | XP_018024592.1/6.4e-176/PREDICTED: insulin-like growth factor 2 mRNA-binding protein 1 isoform X1 [*Hyalella azteca*] |
| CL1257.Contig3 | *Irs* | Insulin receptors | 0.01 | 0.38 | 5.248 | 7.87E-12 | 5.06E-11 | Up | KFM70134.1/7.2e-71/Insulin receptor substrate 2-B, partial [*Stegodyphus mimosarum*] |
| CL2395.Contig2 | *Nos* | NANOS | 4.15 | 9.06 | 1.126 | 6.17E-06 | 2.55E-05 | Up | BAG14279.1/5.5e-10/nanos [*Botryllus primigenus*] |
| CL309.Contig1 | *PAX7* | Paired Box 7 binding protein 1 | 0.99 | 5.57 | 2.492 | 2.51E-46 | 4.55E-45 | Up | XP_018023323.1/7.6e-206/PREDICTED: PAX3- and PAX7-binding protein 1-like [*Hyalella azteca*] |
| CL350.Contig1 | *PRM1* | Sperm Protamine P1-like | 0.17 | 4.59 | 4.755 | 2.67E-15 | 2.05E-14 | Up | XP_012215088.1/2.4e-12/PREDICTED: sperm protamine P1-like [*Linepithema humile*] |
| Unigene1077 | *Sox13* | SRY-related HMG-box containing transcription factor 13 | 0.65 | 1.58 | 1.281 | 4.37E-02 | 9.62E-02 | - | XP_017779251.1/9.8e-20/PREDICTED: transcription factor Sox-13 [*Nicrophorus vespilloides*] |
| Unigene14136 | *Sox14A* | SRY-related HMG-box containing transcription factor 14A | 0.17 | 0.31 | 0.867 | 4.87E-01 | 6.19E-01 | - | AGV15463.1/1.9e-12/SOX14A [*Eriocheir sinensis*] |
| Unigene2106 | *Sox14B* | SRY-related HMG-box containing transcription factor 14B | 4.26 | 5.42 | 0.347 | 4.17E-03 | 1.18E-02 | - | KRT85334.1/4.3e-49/hypothetical protein AMK59_839 [*Oryctes borbonicus*] |
| CL234.Contig4 | *SPATA20* | Spermatogenesis-associated protein 20 | 0.28 | 1.25 | 2.158 | 1.42E-07 | 6.80E-07 | Up | XP_018026417.1/1.7e-242/PREDICTED: spermatogenesis-associated protein 20-like [*Hyalella azteca*] |
| CL798.Contig2 | *SSRF* | Spermatogonia stem-cell renewal factor | 2.65 | 7.6 | 1.520 | 6.33E-06 | 2.61E-05 | Up | Not available |
| CL2141.Contig6 | *Sxl* | Sex-lethal | 74.26 | 14.19 | −2.388 | 0.00E+00 | 0 | Down | APO14323.1/2.3e-108/Sex-lethal 3 [*Macrobrachium rosenbergii*] |
| CL44.Contig28 | *Tra-2* | Transformer-2 | 5.96 | 14.9 | 1.322 | 3.81E-82 | 1.17E-80 | Up | XP_015610488.1/2.5e-70/PREDICTED: beta-1,3-galactosyltransferase 1-like isoform X3 [*Cephus cinctus*] |
| Unigene13323 | *BMP7* | Bone morphogenetic protein 7 | 1.96 | 1.66 | −0.240 | 3.65E-01 | 5.21E-01 | - | AOZ56940.1/3.0e-124/bone morphogenetic protein 7 [*Scylla paramamosain*] |
| Unigene16940 | *Cdc2* | Cell division cycle 2 | 132.73 | 98.04 | −0.437 | 1.61E-24 | 1.76E-23 | - | AGS56255.1/3.4e-171/Cdc2 [*Penaeus monodon*] |
| Unigene8267 | *GEM* | Gem-associated Protein 2-like Isoform X1 | 35.56 | 24.05 | −0.564 | 8.07E-12 | 5.19E-11 | - | XP_013380681.1/5.0e-45/PREDICTED: gem-associated protein 2-like isoform X1 [*Lingula anatina*] |
| CL973.Contig2 | *Gfra2* | GDNF Family Receptor Alpha 2 | 2.89 | 2.29 | −0.336 | 4.65E-02 | 1.01E-01 | - | XP_020787359.1/1.1e-45/zinc finger protein 208-like [*Boleophthalmus pectinirostris*] |
| Unigene10264 | *GnRHr* | Gonadotropin-releasing hormone receptor | 1.81 | 0.75 | −1.271 | 3.12E-01 | 4.58E-01 | - | XP_003392429.1/8.1e-31/hypothetical protein, unlikely [*Leishmania infantum* JPCM5] |
| CL1948.Contig4 | *HSP90* | heat shock protein 90 | 1030.17 | 730.4 | −0.496 | 0.00E+00 | 0.00E+00 | - | ACO83357.1/0.0e+00/heat shock protein 90 [*Penaeus monodon*] |
| Unigene4692 | *Pcna* | Proliferating cell nuclear antigen | 157.7 | 93.45 | −0.755 | 4.76E-78 | 1.39E-76 | - | AOS87743.1/8.7e-143/proliferating cell nuclear antigen *[Penaeus monodon*] |
| Unigene13477 | *Sox5* | SRY-related HMG-box containing transcription factor 5 | 3.07 | 2.17 | −0.501 | 4.18E-04 | 1.39E-03 | - | XP_018008898.1/1.0e-86/PREDICTED: transcription factor Sox-5-like isoform X1 [*Hyalella azteca*] |
| Unigene100 | *Sox6* | SRY-related HMG-box containing transcription factor 6 | 8.96 | 7.75 | −0.209 | 4.26E-02 | 9.40E-02 | - | CDQ73166.1/8.5e-13/unnamed protein product [*Oncorhynchus mykiss*] |
| Unigene6812 | *Sox8* | SRY-related HMG-box containing transcription factor 8 | 1.34 | 0.01 | −0.186 | 9.56E-01 | 9.85E-01 | - | XP_018027588.1/1.8e-41/PREDICTED: transcription factor SOX-8-like [*Hyalella azteca*] |
| CL5203.Contig1 | *Sox9* | SRY-related HMG-box containing transcription factor 9 (promoter region) | 1.34 | 0.01 | −7.066 | 2.35E-01 | 3.86E-01 | - | Not available |
| Unigene17468 | *SPATA5* | Spermatogenesis-associated protein 5 | 17.78 | 9.38 | −0.923 | 1.99E-22 | 2.05E-21 | - | XP_010743648.2/2.1e-111/PREDICTED: spermatogenesis-associated protein 5-like protein 1 [*Larimichthys crocea*] |
| unigene4507 | *Sry* | Sex-determining region Y | 0.49 | 0.09 | −2.445 | 7.19E-05 | 2.64E-04 | Down | XP_013351614.1/2.7e-19/hypothetical protein EMH_0046400 [*Eimeria mitis*] |
| Unigene10779 | *Tra-2c* | Transformer-2c | 4.17 | 1.64 | −1.346 | 8.47E-13 | 5.74E-12 | Down | AFU60542.1/1.1e-138/transformer-2c [*Fenneropenaeus chinensis*] |
| Unigene17633 | *VASA* | DEAD box family of RNA helicases | 281.06 | 218.6 | −0.363 | 1.18E-89 | 3.97E-88 | - | ABQ00071.1/0.0e+00/VASA [*Fenneropenaeus chinensis*] |
